# Efficacy and safety of first-line therapies for persistent, recurrent, or metastatic cervical cancer: a systematic review and exploratory network meta-analysis of immunotherapy

**DOI:** 10.3389/fimmu.2026.1789532

**Published:** 2026-04-16

**Authors:** Xiaoge Wang, Yanxiao Zhang, Chaojun Wang, Li Huang, Zimeng Huang, Guojun Sun

**Affiliations:** 1 College of Pharmacy, Zhejiang University of Technology, Hangzhou, Zhejiang, China; 2 Hangzhou Aeronautical Sanatorium for Special Service of Chinese Air Force, Hangzhou, Zhejiang, China

**Keywords:** immune checkpoint inhibitors, pembrolizumab, cadonilimab, atezolizumab, bevacizumab, persistent/recurrent/metastatic cervical cancer, network meta-analysis

## Abstract

**Background:**

The first-line treatment for persistent, recurrent, or metastatic cervical cancer continues to pose significant clinical challenges. While the application of immune checkpoint inhibitors (ICIs) has transformed the existing treatment paradigm, the lack of direct comparisons between the different immunotherapy regimens limits the selection of optimal strategies for diverse patient populations.

**Methods:**

We have searched through the PubMed, Embase, and Web of Science databases for phase III randomized controlled trials (RCTs) for persistent, recurrent, or metastatic cervical cancer. The primary endpoints were overall survival (OS) and progression-free survival (PFS). The objective response rate (ORR) and the incidence of grade ≥3 treatment-related adverse events (TRAEs) were the secondary endpoints. Data were analyzed using a frequentist network meta-analysis and Bucher’s indirect comparison method.

**Results:**

Four phase III RCTs (1,924 patients) were enrolled. Combinations with bevacizumab, pembrolizumab, and atezolizumab demonstrated comparable efficacy to each other [OS relative hazard ratio (HR) = 1.11, 95%CI = 0.77–1.62; PFS relative HR = 1.09, 95%CI = 0.78–1.52), both significantly superior to chemotherapy. In the treatment setting without bevacizumab, cadonilimab outperformed pembrolizumab plus chemotherapy for OS (relative HR = 0.75, 95%CI = 0.45–1.25) and PFS (relative HR = 0.64, 95%CI = 0.40–1.03). Patients with programmed death-ligand 1 (PD-L1)-positive tumors [combined positive score (CPS) ≥ 1] derived clear benefit from immunotherapy, whereas the PD-L1-negative (CPS < 1) populations experienced limited improvements overall. However, cadonilimab showed a trend toward PFS improvement in this subgroup (HR = 0.65, 95%CI = 0.42–1.03, *p* = 0.06) and reached significance for PFS in the metastatic subgroup (HR = 0.70, 95%CI = 0.54–0.92, *p* < 0.05). The ORR evaluation confirmed that all the combinations with ICIs greatly increased the tumor response rate. In addition, the risk of grade ≥3 TRAEs was comparable between the pembrolizumab and cadonilimab regimens (OR = 1.07, 95%CI = 0.57–2.02).

**Conclusion:**

In the bevacizumab combination setting, pembrolizumab and atezolizumab demonstrate equivalent efficacy. Cadonilimab excels in settings without bevacizumab, showing efficacy in PD-L1-negative patients and metastatic subgroups. Thus, clinical decision-making should integrate multiple pieces of information such as the PD-L1 expression status and bevacizumab eligibility, and individual safety profiles should be considered to create personalized treatment routes. In the future, head-to-head comparative studies are urgently needed to precisely identify optimal treatment regimens for certain types of patient subgroups.

**Systematic Review Registration:**

https://www.crd.york.ac.uk/PROSPERO/view/CRD420251158152, identifier CRD420251158152.

## 1 Introduction

Cervical cancer is the fourth most common tumor in women throughout the world (1). The first-line treatment for persistent, recurrent, or metastatic cervical cancer is still difficult because it is rarely curative and it is frequently hard to avoid drug resistance (2). However, recent clinical trials have demonstrated effectiveness with the immune checkpoint inhibitor (ICI) combination regimens in persistent, recurrent, or metastatic cervical cancer, substantially reshaping the therapeutic landscape (3).

The first-line treatment journey for persistent, recurrent, or metastatic cervical cancer began with GOG 240 (4, 5). This study showed that bevacizumab plus chemotherapy significantly improved patient survival outcomes. Subsequently, the phase II KEYNOTE-158 study confirmed the efficacy of pembrolizumab in previously treated patients with tumor programmed death-ligand 1 (PD-L1) (6). The main phase III KEYNOTE-826 study further advanced progress in the field of first-line immunotherapy. Regardless of whether bevacizumab was used in combination, pembrolizumab had significant efficacy and tolerable adverse reaction in all-comers (7, 8).

Several significant clinical trials have reported first-line immunotherapy plans for individuals with persistent, recurrent, or metastatic cervical cancer. In 2024, the phase III randomized controlled trial (RCT) BEATcc was conducted to investigate the effect of atezolizumab plus chemotherapy and bevacizumab (9). The phase III RCT COMPASSION-16 was conducted to study the effects of cadonilimab plus chemotherapy (±bevacizumab) (10). Both phase III studies successfully achieved their primary endpoints and showed different degrees of survival benefits. In addition, a number of phase II trials have been quite impressive. Among these combinations, the programmed cell death protein 1 (PD-1) inhibitor QL1604 plus chemotherapy achieved an objective response rate (ORR) of 58.7%, with progression-free survival (PFS) of 8.1 months. Patients who have high PD-L1 expression exhibited prominent response (11). Another phase II study showed the regimen of tislelizumab combined with low-dose bevacizumab and chemotherapy, yielding remarkable results: an ORR of 91.5% and a PFS of 22.6 months (12).

However, there are no head-to-head clinical trials directly comparing different immunotherapies. Bevacizumab offers survivors, but also carries toxicity risks (5). Due to differences in the PD-L1 expression levels between patients and the limited suitability of bevacizumab for some individuals, it remains unclear which treatment regimen is more effective for specific patient groups (13). Consequently, there is a great deal of ambiguity regarding which treatment plan to use in clinical practice.

Therefore, we conducted a systematic review and network meta-analysis to explore treatment gaps and included the overall phase III RCTs in the first-line treatment of persistent, recurrent, or metastatic cervical cancer. These trials evaluated novel treatment regimens, including programmed cell death protein 1 (PD-1)/cytotoxic T-lymphocyte-associated protein 4 (CTLA-4) bispecific antibodies. To explore relevant subgroups, we performed indirect comparisons to assess treatment efficacy in clinically relevant patient populations. Given the lack of direct comparative trials, we further investigated stratification factors, such as the PD-L1 expression status and concurrent bevacizumab use.

This study aimed to preliminarily assess the relative efficacy of different immunotherapeutic strategies and develop testable hypotheses for which strategy may be the most effective for specific patients. The study findings emphasize the urgent need for well-designed head-to-head RCTs and highlight the necessity of establishing evidence-based treatment decision-making processes.

## 2 Methods

The protocol for this systematic review has been registered in the PROSPERO database and assigned the identifier CRD420251158152. After the execution of the present research, the final reporting of its findings was conducted in strict accordance with the Preferred Reporting Items for Systematic Reviews and Meta-Analyses (PRISMA) statement principles (**Supplementary Table S1**) (14).

### 2.1 Search strategy and study identification

A systematic search was performed in the Embase, PubMed, and Web of Science databases. This study included all records from the beginning until December 29, 2025, using a strategy of keywords such as persistent, recurrent, or metastatic cervical cancer; chemotherapy; bevacizumab; ICIs; and phase III. The exact search string is provided in **Supplementary Table S2**. In addition, manual searches of the reference lists from other reviews and conference proceedings were done to determine any studies missed by the electronic search.

### 2.2 Eligibility criteria

The study included phase III RCTs of ICIs (monotherapy or combination). All included RCTs had a control arm of standard chemotherapy (with/without bevacizumab). We excluded phase I trials, phase II trials, non-English publications, duplicate reports, and non-interventional studies.

### 2.3 Study selection and data extraction

For this study, two researchers independently performed the literature screening. Any discrepancy that showed up through this procedure was resolved with a third researcher. Furthermore, the data extracted from the RCTs were included in accordance with a pre-arranged tabular table, encompassing the following items: a) study information; b) study design and baseline characteristics; c) intervention and control details; d) efficacy outcomes (PFS, OS, and ORR); e) safety outcomes [grade ≥3 adverse events (AEs)]; and f) biomarker and subgroup data.

### 2.4 Risk of bias assessment

A quality appraisal for the method of each RCT entered into the network meta-analysis was performed. This assessment of potential bias used the Cochrane risk-of-bias 2.0 (RoB 2.0) instrument (15).

### 2.5 Statistical analysis

This work carried out the study with a network meta-analysis based on a frequency framework and performed indirect comparisons using the Bucher method. The primary estimates included both fixed-effects and random-effects models. The *I*^2^ statistic and Cochran’s *Q* test were used to quantitatively assess statistical heterogeneity, and a test for inconsistency was performed. Ranking of treatment efficacy was achieved by finding the sum of areas using the surface under the cumulative ranking (SUCRA) curve. To verify the statistical appropriateness of the use of hazard ratios (HRs) as the primary effect measure, the proportional hazards (PH) assumption was formally assessed for the overall population of all included trials via Schoenfeld residual tests based on reconstructed individual patient data (IPD) from published Kaplan–Meier curves. Exploratory stratified analyses were conducted for different subgroups such as the PD-L1 expression levels and the bevacizumab combination therapy. The findings should be considered hypothesis-generating rather than definitive. All statistical analyses were performed with R (version 4.4.5).

## 3 Results

### 3.1 Literature search and study selection

**Figure 1:**
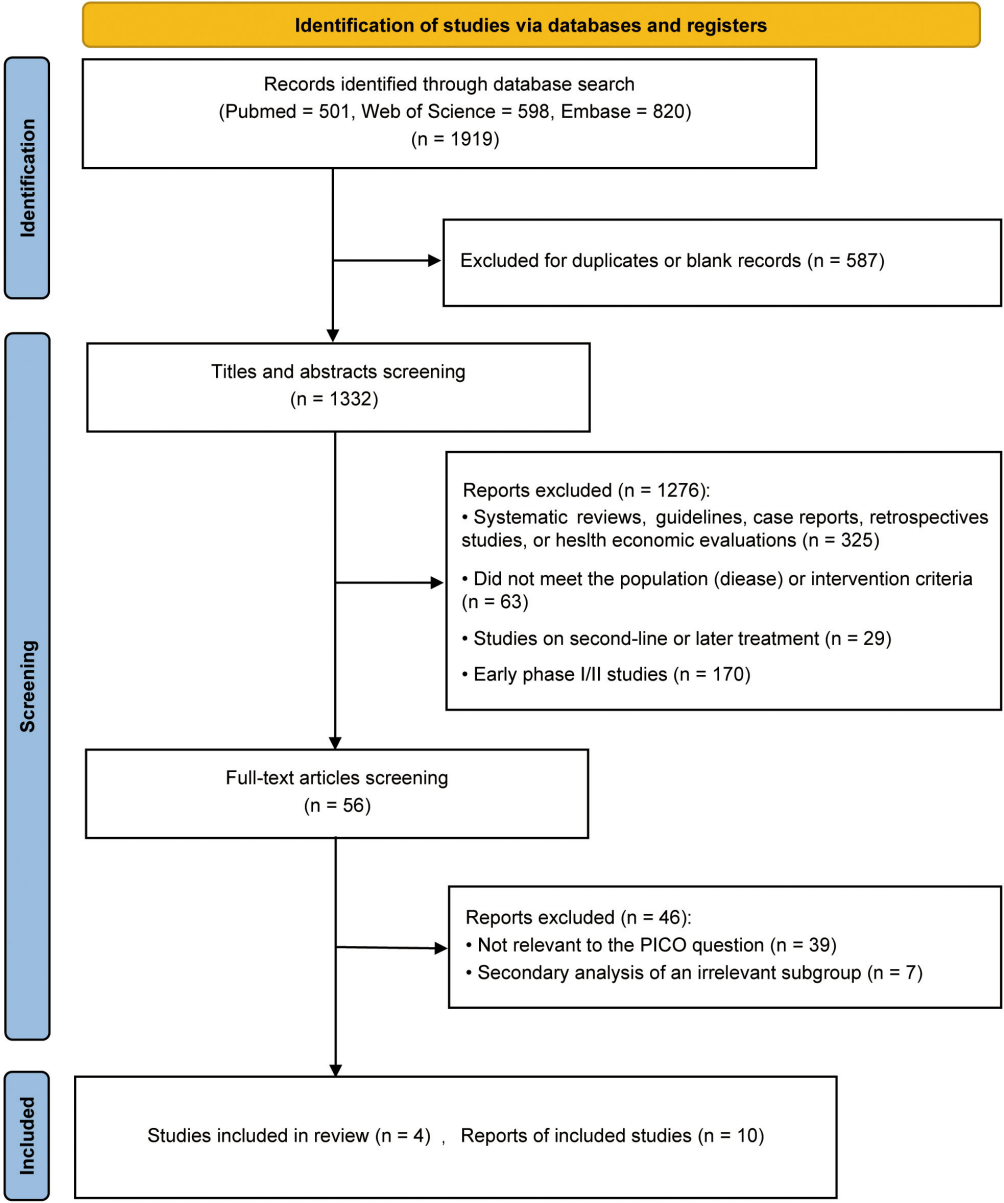
Preferred reporting items for systematic reviews and meta-analyses (PRISMA) flow diagram of study selection.

We conducted a systematic search of the Embase, Web of Science, and PubMed databases, yielding a total of 1,919 records. After removing 587 duplicate entries, four phase III RCTs meeting the PICOS criteria were ultimately included. These RCTs comprised a total of nine published articles and one conference abstract, encompassing 1,924 patients (4, 5, 7, 8, 10, 16–20). The detailed screening process is illustrated in the PRISMA flow diagram (**Figure 1**). The basic characteristics and efficacy data of the included RCTs are summarized in **Table 1**, and the results of the risk of bias assessment are presented in **Supplementary Figure S1**. Two of the trials were assessed as having “some concerns” with regard to risk of bias due to their open-label design. The absence of blinding may have introduced bias, potentially affecting the investigator’s assessment of the endpoints.

**Table 1:** Characteristics of the included clinical trials.

Study	Study design	Sample size	Intervention *vs*. control	Primary outcomes (efficacy) (intervention *vs*. control)	Safety (intervention *vs*. control)	PD-L1 status and key subgroups
GOG240 (4, 5)	Open-label, multicenter	452	Chemotherapy + bevacizumab *vs*. Chemotherapy	ORR = 48.9% *vs*. 35.6% PFS: 8.2 *vs*. 6.0 months OS: 16.8 *vs*. 13.3 months	Grade ≥2 hypertension: 25% *vs*. 2% Grade ≥3 thromboembolism: 8% *vs*. 1% Grade ≥3 fistula: 6% *vs*. <1%	Not reported OS benefit consistent across all key subgroups
KEYNOTE-826 (7, 8)	Double-blind, placebo-controlled	617	Pembrolizumab ± bevacizumab + chemotherapy *vs*. Placebo ± bevacizumab + chemotherapy	ORR = 66.2% *vs*. 51.5% PFS: 10.4 *vs*. 8.2 months OS: 26.4 *vs*. 16.8 months	Grade ≥3 AEs: 82.4% *vs*. 75.4% imAEs: 34.5% *vs*. 16.5%	PD-L1 CPS: <1 (~11%), 1–9 (~37%), ≥10 (~52%) Efficacy benefit consistent across PD-L1+ and all-comer populations, regardless of bevacizumab use
BEATcc (19)	Open-label	410	Atezolizumab + bevacizumab + chemotherapy *vs*. Bevacizumab + chemotherapy	ORR = 84% *vs*. 72% PFS: 13.7 *vs*. 10.4 months OS: 32.1 *vs*. 22.8 months	Grade ≥3 AEs: 79% *vs*. 75%	All-comers (no biomarker selection) 80% squamous histology Efficacy benefits favored the experimental group across all pre-specified subgroups.
COMPASSION-16 (10)	Double-blind, placebo-controlled	445	Cadonilimab ± bevacizumab + chemotherapy *vs*. Placebo ± bevacizumab + chemotherapy	ORR = 83% *vs*. 69% PFS: 12.7 *vs*. 8.1 months OS: Not reached *vs*. 22.8 months	Grade ≥3 AEs: 85% *vs*. 80% imAEs: 46%	PD-L1−: 28% cadonilimab *vs*. 24% placebo PD-L1+: 72% cadonilimab *vs*. 76% placebo.

ORR, objective response rate; PFS, progression-free survival; OS, overall survival; AE, adverse event; imAE, immune-mediated adverse event; PD-L1 CPS, programmed death-ligand 1 combined positive score; PD-L1+, PD-L1-positive; PD-L1−, PD-L1-negative.

### 3.2 Network meta-analysis of OS and PFS

Given the clinical variation in bevacizumab combination regimens across the BEATcc, KEYNOTE-826, and COMPASSION-16 trials, data from KEYNOTE-826 and COMPASSION-16 were stratified according to bevacizumab use. By including the phase III GOG 240 trial, a comprehensive evidence network comprising seven treatment regimens was established (**Supplementary Figure S2A**). Construction of this strategic network was specifically intended to enable indirect “head-to-head” comparisons between different ICI backbones using chemotherapy as the common comparator. Due to the star-shaped, open structure of the network and the fact that each intervention comparison was derived from a single study, quantitative assessment of heterogeneity (Cochran’s *Q* and *I*^2^) and formal inconsistency testing could not be performed. Global Schoenfeld residual tests based on reconstructed IPD demonstrated that the PH assumption was adequately met for the major included trials (all global *p*-values >0.05) (**Supplementary Table S3**). Furthermore, as the point estimates for HRs are mathematically identical between the fixed-effects and random-effects models in the absence of closed loops, only the results from the fixed-effects model are presented.

**Figure 2:**
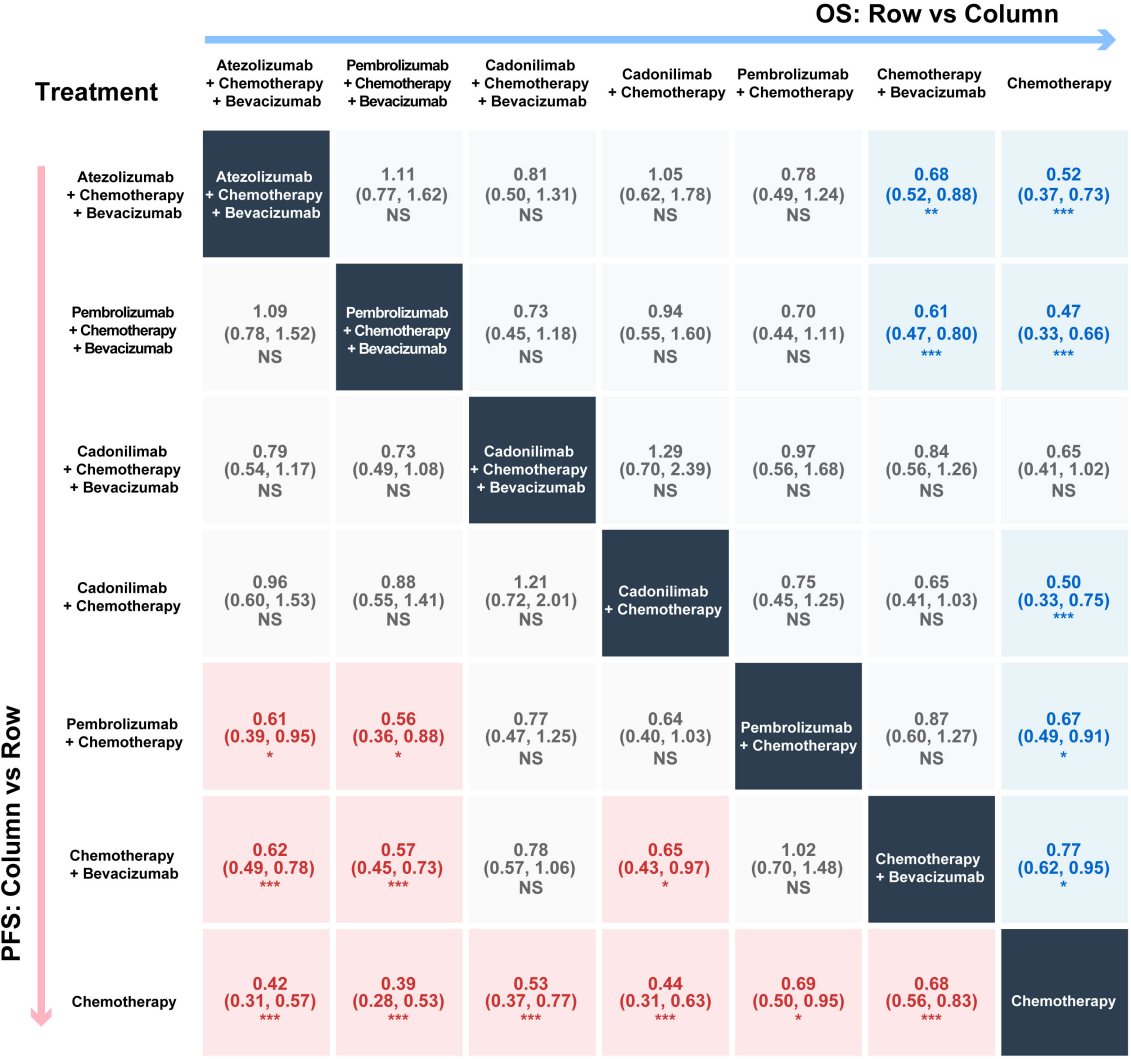
Network meta-analysis: League table for progression-free survival (PFS) and overall survival (OS). The *lower left triangle* presents the results for PFS, with data expressed as hazard ratios (HRs) and 95% confidence intervals (CIs) calculated as column-*versus*-row treatments; *red shading* indicates a statistically significant survival benefit (HR < 1). The *upper right triangle* displays the results for OS (calculated similarly); *blue shading* indicates a statistically significant survival benefit (HR < 1). Statistical significance is indicated as: ***p < 0.001; **p < 0.01; *p < 0.05. *NS*, not significant.

The network meta-analysis showed that majority of the immune combination therapies outperformed chemotherapy alone, leading to improved patient survival outcomes, although the statistical significance varied across different regimens. For OS (**Figure 2**), among the regimens combining bevacizumab and chemotherapy, the pembrolizumab regimen demonstrated the best efficacy (HR = 0.47, 95%CI = 0.33–0.66), followed by the atezolizumab combination (HR = 0.52, 95%CI = 0.37–0.73). In contrast, the combination of cadonilimab with bevacizumab and chemotherapy showed a trend toward benefit, but did not reach statistical significance (HR = 0.65, 95%CI = 0.41–1.02). Head-to-head indirect comparisons revealed that atezolizumab and pembrolizumab were highly comparable (relative HR = 1.11, 95%CI = 0.77–1.62), with both showing numerically superior efficacy compared with the cadonilimab combination regimen (relative HRs of 0.81 and 0.73, respectively), although these differences did not reach statistical significance. Notably, in the bevacizumab-free subgroup, cadonilimab plus chemotherapy demonstrated superior performance compared with chemotherapy alone (HR = 0.50, 95%CI = 0.33–0.75), even surpassing pembrolizumab plus chemotherapy (HR = 0.67, 95%CI = 0.49–0.91). Further indirect comparisons suggested that, when combined with chemotherapy alone, the bispecific antibody regimens hold greater potential for survival benefit compared with the monoclonal antibody regimen (relative HR = 0.75, 95%CI = 0.45–1.25).

The pattern for PFS mirrored that of OS (**Figure 2**). Among the quadruplet regimens (combined with bevacizumab and chemotherapy), both pembrolizumab (HR = 0.39, 95%CI = 0.28–0.53) and atezolizumab (HR = 0.42, 95%CI = 0.31–0.57) therapies maintained a significant and comparable advantage, with no statistical difference between them (relative HR = 1.09, 95%CI = 0.78–1.52). The head-to-head indirect comparison results showed that both the pembrolizumab (relative HR = 0.73, 95%CI = 0.49–1.08) and atezolizumab (relative HR = 0.79, 95%CI = 0.54–1.17) combination therapies outperformed the cadonilimab combination therapy, although the results did not demonstrate statistical significance. Conversely, in the bevacizumab-free subgroup, the comparative efficacy hierarchy shifted significantly. Cadonilimab plus chemotherapy remained highly effective compared with chemotherapy alone (HR = 0.44, 95%CI = 0.31–0.63). Indirect comparisons revealed a numerical advantage for cadonilimab over pembrolizumab plus chemotherapy (relative HR = 0.64, 95%CI = 0.40–1.03). The forest plot for the network meta-analysis is shown in **Supplementary Figure S3**.

### 3.3 Subgroup analysis of OS and PFS

Subgroup analyses for the PD-L1 expression status and metastatic disease were conducted using the Bucher method for indirect comparisons (**Supplementary Tables S4**, **S5**).

In patients with PD-L1 combined positive score (CPS) < 1, neither immunotherapy combination showed significant survival benefits. For OS, pembrolizumab and cadonilimab combined with chemotherapy ± bevacizumab yielded HRs of 0.87 (95%CI = 0.50–1.52) and 0.77 (95%CI = 0.44–1.34), respectively (*p* > 0.05). Similar results were observed for PFS. Although cadonilimab showed a trend toward improvement (HR = 0.65, 95%CI = 0.42–1.03, *p* = 0.060), this did not achieve statistical significance. Indirect comparisons between the two regimens revealed no significant differences in either OS or PFS.

In contrast, patients with PD-L1 CPS ≥ 1 derived substantial benefit from immunotherapy combinations. Pembrolizumab plus chemotherapy ± bevacizumab achieved an HR of 0.60 (95%CI = 0.49–0.74, *p* < 0.001) in the PD-L1-negative subgroup, while cadonilimab reached 0.69 (95%CI = 0.49–0.97, *p* < 0.05). Both regimens also demonstrated significant efficacy for PFS, with HRs of 0.58 (95%CI = 0.47–0.71) and 0.62 (95%CI = 0.47–0.83), respectively. Indirect comparisons showed comparable efficacy between the two regimens (OS: HR = 0.87, *p* = 0.491; PFS: HR = 0.94, *p* = 0.708).

In the metastatic disease subgroup, cadonilimab plus chemotherapy ± bevacizumab approached significance for OS (HR = 0.73, 95%CI = 0.52–1.02, *p* ≈ 0.05) and reached statistical significance for PFS (HR = 0.70, 95%CI = 0.54–0.92, *p* < 0.05). Pembrolizumab failed to achieve significance for either endpoint. Indirect comparisons indicated similar efficacy between the two regimens (*p* > 0.05).

Notably, the recently reported East Asian subgroup from KEYNOTE-826 demonstrated superior efficacy compared with the overall population (18). For OS, the East Asian subgroup achieved an HR of 0.53 (95%CI = 0.28–0.99), whereas the overall population showed an HR of 0.63 (95%CI = 0.52–0.77). Similarly, for PFS, the East Asian subgroup had an HR of 0.42 (95%CI = 0.23–0.77) *versus* an HR of 0.61 (95%CI = 0.50–0.74) in the overall population. In comparison, COMPASSION-16 enrolled exclusively Chinese patients. The OS and PFS results from this all-Chinese cohort were HR = 0.65 (95%CI = 0.49–0.87) and HR = 0.62 (95%CI = 0.49–0.79), respectively. These findings closely aligned with the overall population data from KEYNOTE-826.

### 3.4 ORR and safety analysis

**Figure 3:**
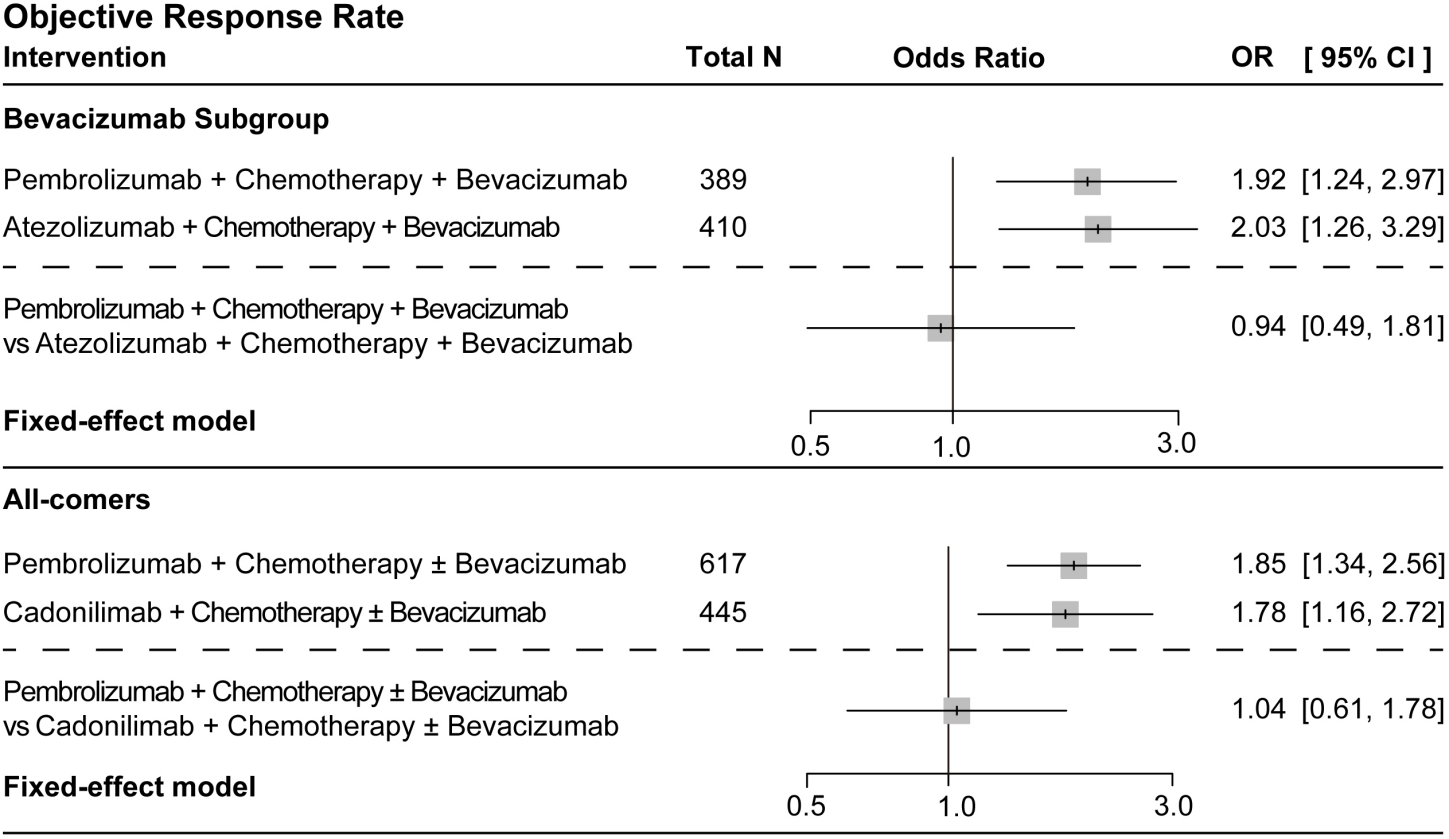
Forest plots of the objective response rate in the bevacizumab subgroup and the all-comer population. The forest plots present the odds ratios (ORs) with 95% confidence intervals (CIs) for efficacy comparisons. For the bevacizumab subgroup, the immunotherapy combination regimen was compared with the standard control group receiving chemotherapy plus bevacizumab. For the all-comers, the intervention was evaluated based on the control group receiving chemotherapy (with or without bevacizumab). All analyses were conducted using a fixed-effects model. An OR > 1 indicates higher odds of response for the experimental immunotherapy regimen *versus* its control. *CI*, confidence interval; *OR*, odds ratio.

Since the COMPASSION-16 trial has not yet reported the ORR data for the bevacizumab subgroup, only the all-comer population from this study was included in the analysis. ORR was indirectly compared in separate three-regimen networks, as shown in **Supplementary Figures S2B**, **C**. The ORR analysis revealed that all immunotherapy combinations outperformed chemotherapy alone. In the bevacizumab subgroup, pembrolizumab (OR = 1.92, 95%CI = 1.24–2.97) and atezolizumab (OR = 2.03, 95%CI = 1.26–3.29) combined with chemotherapy and bevacizumab both significantly improved the ORRs, with similar efficacy between them (OR = 0.94, 95%CI = 0.49–1.81) (**Figure 3**). In the all-comer population, pembrolizumab (OR = 1.85, 95%CI = 1.34–2.56) and cadonilimab (OR = 1.78, 95%CI = 1.16–2.72) combined with chemotherapy ± bevacizumab also significantly enhanced the ORRs, showing no significant difference between regimens (OR = 1.04, 95%CI = 0.61–1.78) (**Figure 3**).

**Figure 4:**
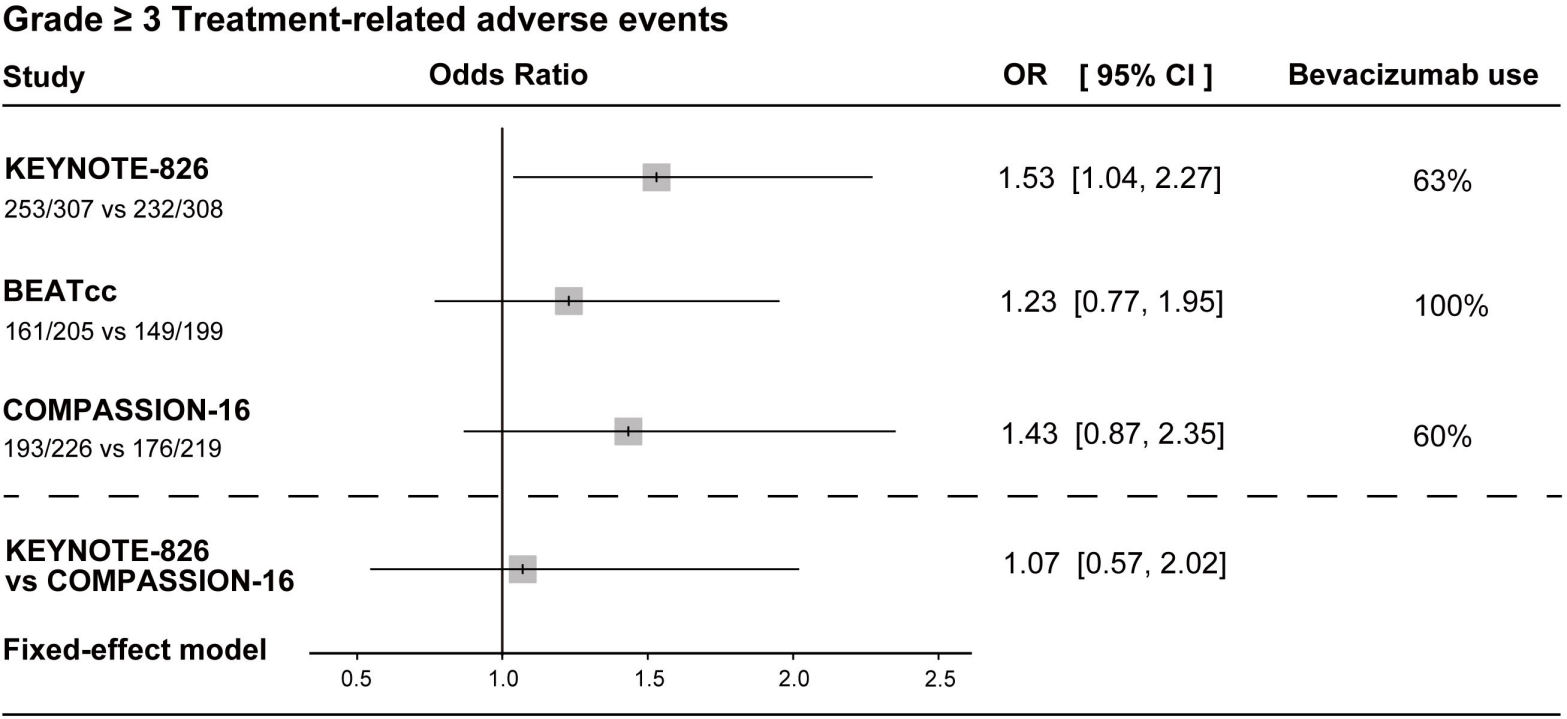
Forest plots of grade ≥3 treatment-related adverse events. The plots present the odds ratios (ORs) with 95% confidence intervals (CIs) for safety comparisons. The analysis was conducted using a fixed-effects model. An OR > 1 indicates higher odds of adverse events for the experimental immunotherapy regimen *versus* its control. CI, confidence interval; OR, odds ratio.

With regard to safety, all three included RCTs reported grade ≥3 treatment-related AEs (TRAEs) (**Figure 4**). The KEYNOTE-826 study demonstrated a statistically significant increase in severe toxicities (OR = 1.53, 95%CI = 1.04–2.27), whereas the BEATcc (OR = 1.23, 95%CI = 0.77–1.95) and COMPASSION-16 (OR = 1.43, 95%CI = 0.87–2.35) studies did not reach conventional statistical significance. The indirect comparison based on the Bucher method further revealed a high degree of similarity in the risk of severe toxicities between the pembrolizumab and cadonilimab combination regimens (OR = 1.07, 95%CI = 0.57–2.02). In addition, 10% of the patients in the cadonilimab combination regimen experienced grade 3–4 immune-related AEs (irAEs), numerically lower than the 12% reported for the pembrolizumab-based combination regimen in KEYNOTE-826 (10, 16).

## 4 Discussion

This study conducted a network meta-analysis to integrate and compare the results from the KEYNOTE-826, BEATcc, and COMPASSION-16 trials, revealing the efficacy and safety profiles of different ICI regimens. In the absence of head-to-head comparative data, this research specifically evaluated the performance differences between monoclonal antibodies (pembrolizumab and atezolizumab) and bispecific antibodies (cadonilimab) in the context of combination therapy with or without bevacizumab.

The results from the network meta-analysis indicate that the addition of bevacizumab to immunotherapy regimens with pembrolizumab significantly enhances efficacy (8). Although this difference can be caused by the baseline features of the patients in the different subgroups, the synergistic effect of immune-angiogenesis inhibitors is attributed to many mechanisms. Bevacizumab can improve abnormal vascular structures and indirectly enhance the antitumor immune responses by reducing the activity of inhibitory immune factors in tumor cells, thereby creating a more favorable microenvironment for the efficacy of ICIs (21).

Notably, in COMPASSION-16, cadonilimab plus chemotherapy (without bevacizumab) demonstrated a much better efficacy compared with the cadonilimab plus chemotherapy plus bevacizumab treatment regimen (OS: HR = 0.50 *vs*. 0.84; PFS: HR = 0.44 *vs*. 0.78). The opposite result was observed in combination regimens with pembrolizumab (OS: HR = 0.67 *vs*. 0.61; PFS: HR = 0.69 *vs*. 0.57), which may be related to the distinct mechanism of action of cadonilimab as a bispecific antibody. Cadonilimab can simultaneously target both PD-1 and CTLA-4 checkpoints, enhancing the efficacy of immunotherapy by synergistically promoting T-cell activation (22). Furthermore, for patients with underlying conditions or bleeding risks who are unsuitable for bevacizumab, previous single-target ICI plus chemotherapy regimens failed to achieve 2-year OS (8). In contrast, cadonilimab plus chemotherapy demonstrated a median OS of 28.2 months (20). This finding suggests that the bispecific antibody maintains potent antitumor activity as a backbone therapy, even in the absence of anti-angiogenic synergy.

Although the results were primarily good overall, the different groups of patients had noticeable results as well. The PD-L1 expression status is an important indicator of treatment response (13). For patients who were PD-L1-positive (CPS ≥ 1), both the pembrolizumab and cadonilimab regimens derived significant benefits. In the PD-L1-negative (CPS < 1) subgroup, the clinical potential of the two agents showed significant differences. Although the confidence intervals for both treatments included 1, cadonilimab had a strong trend toward benefit in PFS (HR = 0.65, 95%CI = 0.42–1.03), where the upper limit approached significance (*p* = 0.060) compared with pembrolizumab, which did not show a clear benefit in this subgroup (HR ~ 1). Although the difference between the two groups did not reach statistical significance in the indirect comparison, the numerical advantage of cadonilimab in this subgroup suggests that dual PD-1/CTLA-4 inhibition may be more effective than PD-1 inhibition alone in overcoming the immunosuppressive barrier. However, this hypothesis requires confirmation in the head-to-head trials. For the metastatic disease subgroup, cadonilimab also demonstrated a more pronounced benefit signal compared with pembrolizumab, suggesting that cadonilimab may offer a specific advantage for more advanced disease with high tumor burden (10).

ORR analysis further supports the results. Higher response rates translate to improved symptom control and quality of life. Compared with the control, all combination immunotherapy regimens significantly improved the ORR. However, indirect comparisons showed no significant differences in the ORR among regimens, suggesting that immunotherapy offers broadly comparable benefits in symptom control. The safety forest plot indicates that the addition of immunotherapy generally increases the risk of grade ≥3 TRAEs. Specifically, the KEYNOTE-826 trial demonstrated a statistically significant increase in this risk (OR = 1.53, 95%CI = 1.04–2.27). However, our indirect comparison revealed that the risk of grade ≥3 TRAEs is highly comparable between the pembrolizumab and cadonilimab regimens in the overall population (OR = 1.07, 95%CI = 0.57–2.02). Notably, with regard to the incidence of grade 3–4 irAEs, the dual-targeted cadonilimab demonstrated a numerically lower rate than the single-targeted pembrolizumab (10% *vs*. 12%). This safety advantage likely stems from cadonilimab’s mechanism of localized immune activation within the tumor microenvironment (10). By minimizing nonspecific binding to single-positive T cells in normal peripheral tissues, cadonilimab mitigates the risk of severe systemic autoimmune toxicities while fully preserving its potent dual-pathway antitumor efficacy (22).

Moreover, the significant geographical and racial disparities among patients enrolled in different clinical trials further complicate the selection of optimal treatment regimens for patients. The East Asian subgroup in KEYNOTE-826 had a tendency toward better efficacy than all-comers, but with confidence intervals that were much wider than those of all-comers (OS: 0.28–0.99 *vs*. 0.52–0.77; PFS: 0.23–0.77 *vs*. 0.50–0.74). The results from COMPASSION-16, with an exclusively Chinese population, were highly consistent with the all-comer population data from KEYNOTE-826, but discordant with the East Asian subgroup data from KEYNOTE-826. This subgroup had a small sample size and included participants from multiple countries, exhibiting diversity in genetic backgrounds, tumor biology, and clinical practices. As a study designed in China, COMPASSION-16 may have more representative data for Chinese patients. These results highlight the impact of population heterogeneity on cross-study comparisons, necessitating prospective studies in specific regions to validate these observations.

To our knowledge, this is the first network meta-analysis performing an exploratory comparative analysis of three immunosuppressants based on whether bevacizumab was combined. However, the exploration in this study has some limitations. Due to the limited number of phase III trials, the evidence network exhibits a star-shaped open structure without closed loops. Consequently, the point estimates for the HRs remained mathematically identical between the fixed-effects and random-effects models, and formal consistency testing was not feasible. In the absence of head-to-head trials, it was not possible to account for all confounding. The presence of baseline differences may have led to the null hypothesis no longer holding. These discrepancies were most obvious on patient racial makeup and on the use of blinding, which might have impacted the generalizability of the findings, necessitating cautious interpretation.

## 5 Conclusion

It is now considered standard care for recurrent or metastatic cervical cancer to use a combination of immunotherapies. PD-L1 expression is an important predictive biomarker. Cadonilimab has demonstrated potential in PD-L1-negative (CPS < 1) patients and in those not receiving bevacizumab. Pembrolizumab plus bevacizumab is effective in PD-L1-positive (CPS ≥ 1) disease. In real-world practice, bevacizumab eligibility, patient characteristics, and economic considerations must be integrated into the decision-making process.

In the future, further effort should be put into conducting head-to-head trials that are aimed at specific patient groups with a specific emphasis on groups such as PD-L1-negative patients and refractory metastatic individuals. Moreover, other emerging biomarkers such as tumor mutational burden, immune gene expression pattern, and circulating tumor cells must be further explored to determine the best patient groups that can receive the targeted immunotherapy regimens. Finally, it is especially essential to undertake cost-effectiveness studies in low- and middle-income nations, where the cervical cancer burden is the greatest.

## Data Availability

The raw data supporting the conclusions of this article will be made available by the authors, without undue reservation.
