# Supplementary material for: Efficacy and safety of first-line therapies for persistent, recurrent, or metastatic cervical cancer: a systematic review and exploratory network meta-analysis of immunotherapy
Source: Front Immunol. 2026 Apr 16;17:1789532. doi: 10.3389/fimmu.2026.1789532 (PMC13128609; doi:10.3389/fimmu.2026.1789532)
Supplement: Supplementary file 1 [file DataSheet1.docx]

Supplementary Material

# Supplementary Figures

# Figure S1. Risk of bias assessment.


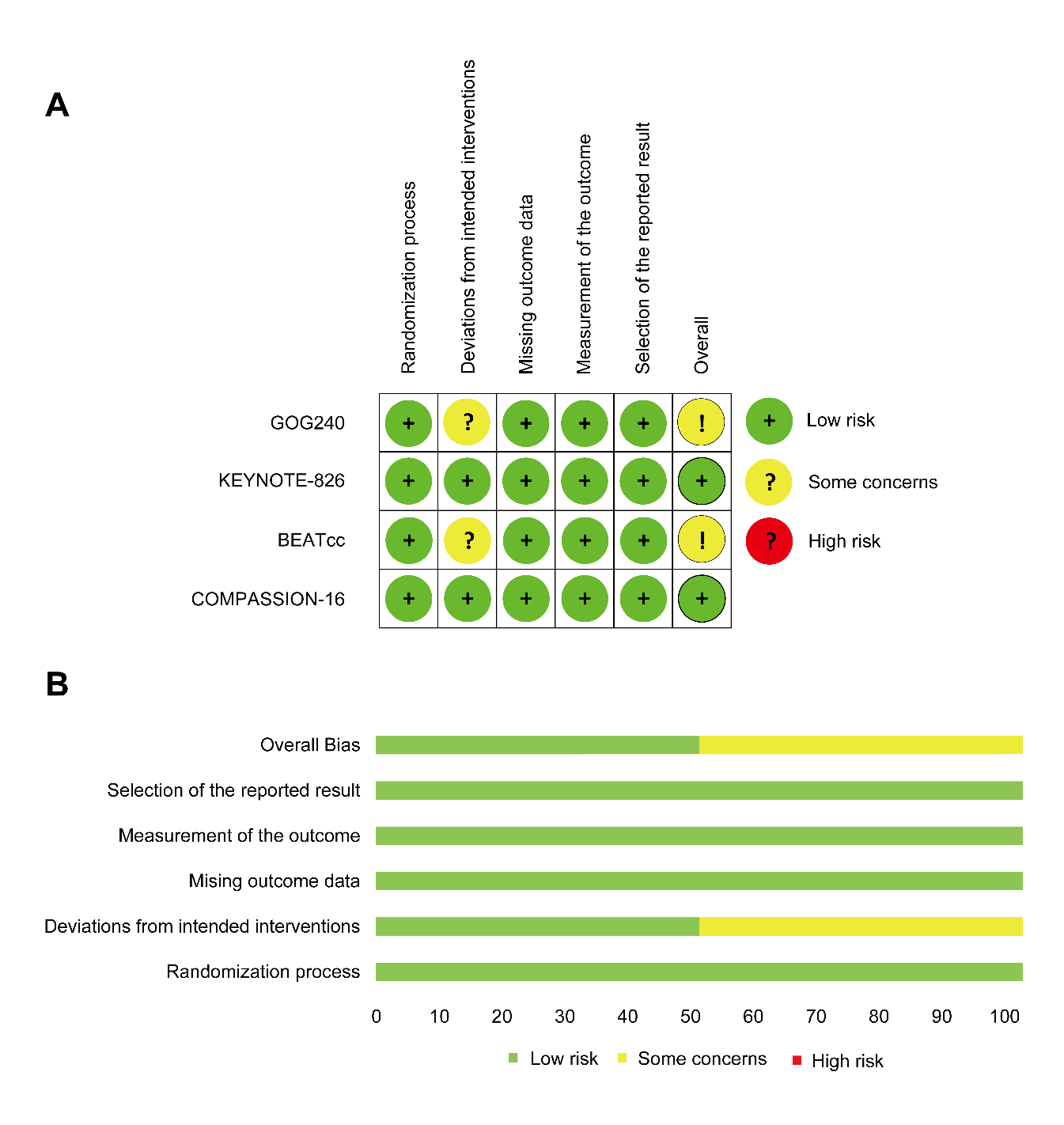


(A) Risk of bias summary: assessments on each risk of bias item for each included study. (B) The assessment of each risk of bias item (low, some concerns, and high) for each included study is expressed as a percentage.

# Figure S2. Network Plots.

**
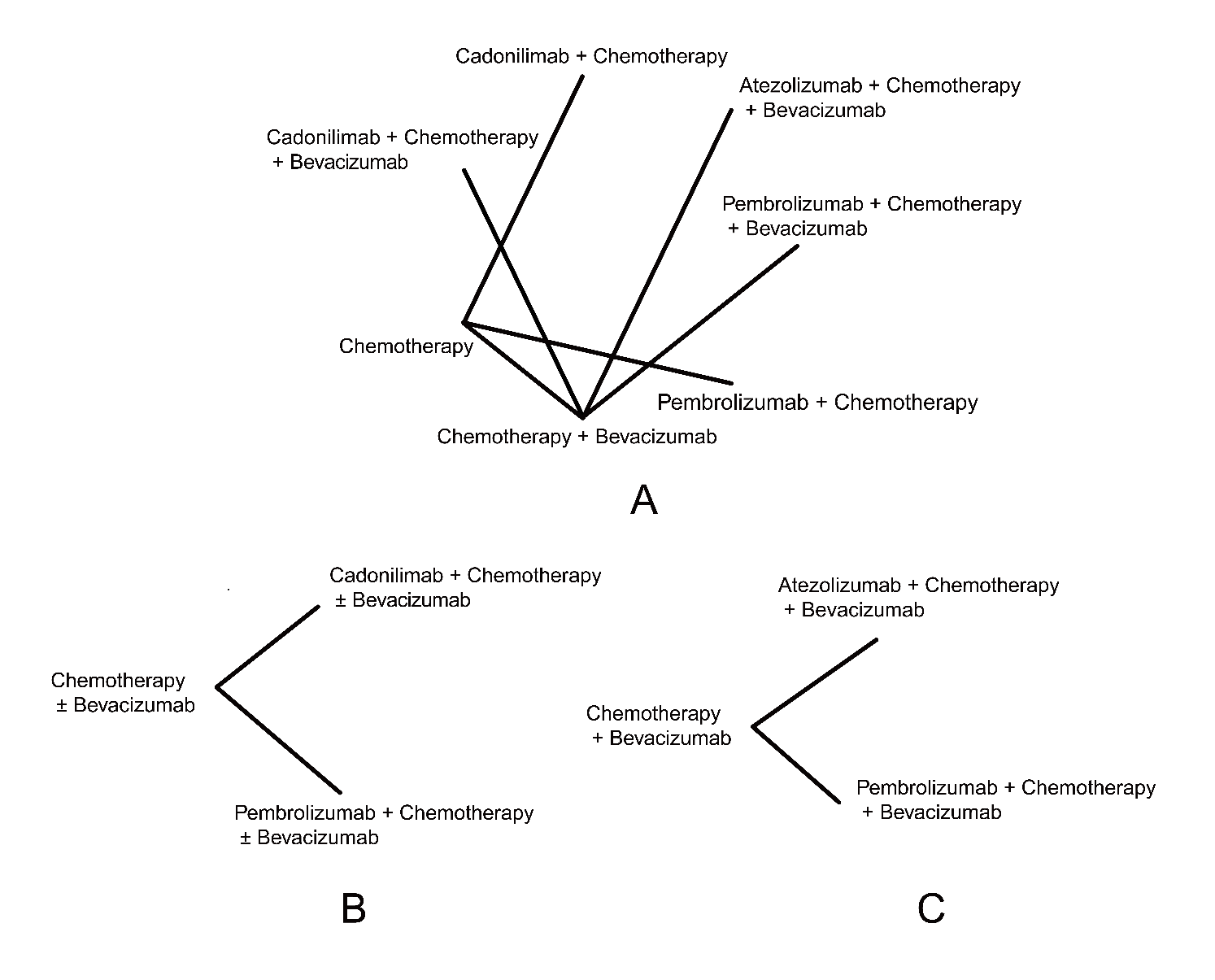
**

(A) Comparison network for overall survival and progression-free survival. (B) Network for objective response rate in the all-comers population (chemotherapy with or without bevacizumab). (C) Network for objective response rate in the bevacizumab-eligible subgroup.

# Figure S3. Forest plots of immunotherapy combinations stratified by bevacizumab eligibility: (A) Overall survival and (B) Progression-free survival.


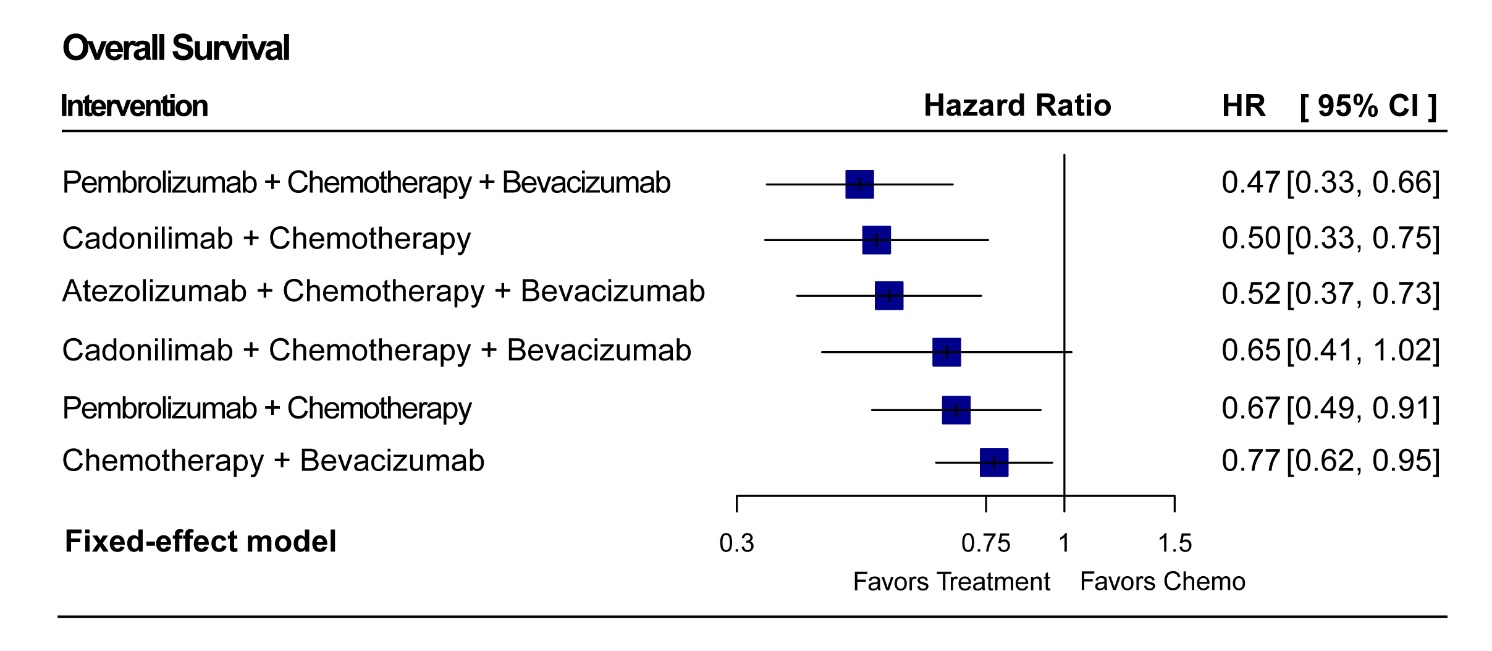


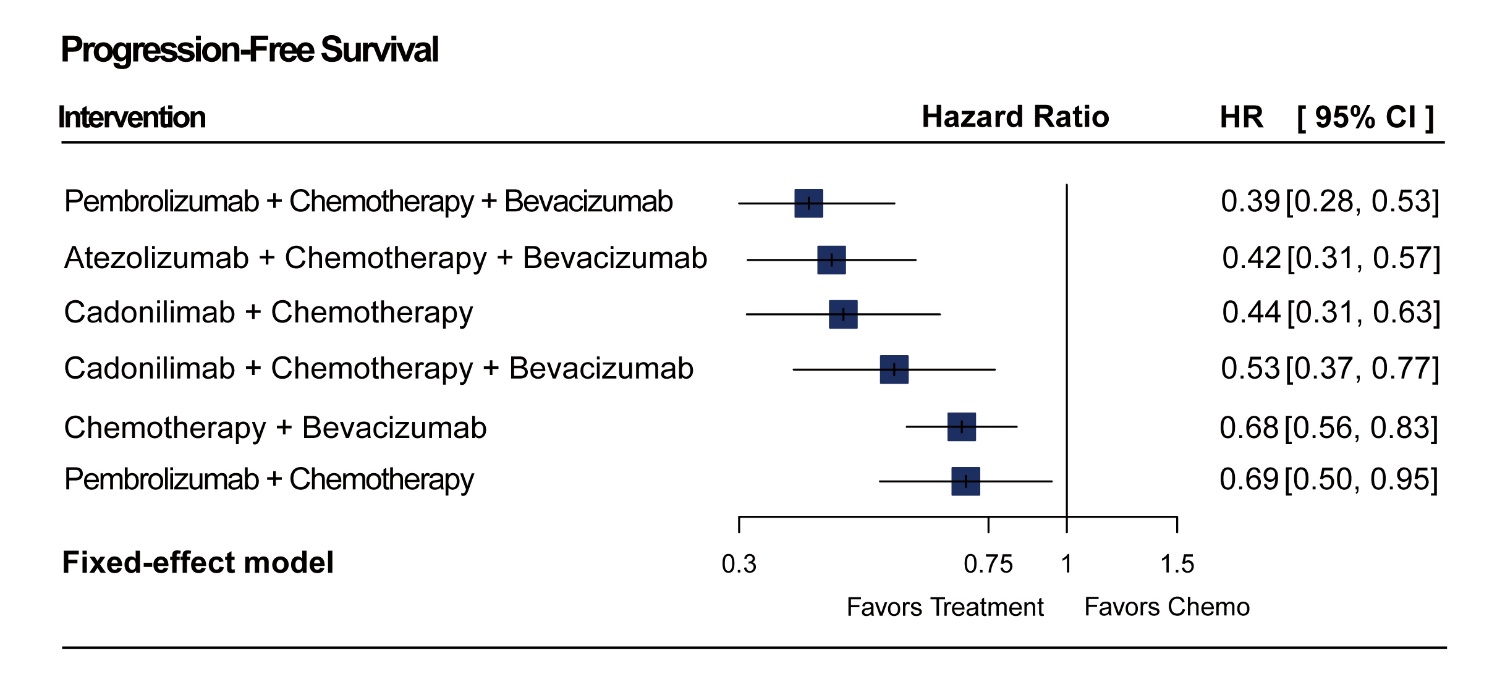


The forest plot presents mixed (direct and indirect) estimates from a frequentist network meta-analysis, with all immunotherapy-based regimens compared against the common reference of chemotherapy alone. The analysis was performed under a fixed-effect model. Treatments are ranked by the point estimate of the hazard ratio (HR) within each outcome. All HRs are presented with 95% confidence intervals (CIs). Abbreviations: CI, confidence interval; HR, hazard ratio.

# Supplementary Tables

# Table S1 PRISMA checklist

| **Section and Topic** | **Item #** | **Checklist item** | **Location where item is reported** |
| --- | --- | --- | --- |
| **TITLE** | | |  |
| Title | 1 | Identify the report as a systematic review. | Title page |
| **ABSTRACT** | | |  |
| Abstract | 2 | See the PRISMA 2020 for Abstracts checklist. | Abstract section |
| **INTRODUCTION** | | |  |
| Rationale | 3 | Describe the rationale for the review in the context of existing knowledge. | introduction |
| Objectives | 4 | Provide an explicit statement of the objective(s) or question(s) the review addresses. | introduction |
| **METHODS** | | |  |
| Eligibility criteria | 5 | Specify the inclusion and exclusion criteria for the review and how studies were grouped for the syntheses. | Methods – Eligibility Criteria |
| Information sources | 6 | Specify all databases, registers, websites, organisations, reference lists and other sources searched or consulted to identify studies. Specify the date when each source was last searched or consulted. | Methods – Search Strategy |
| Search strategy | 7 | Present the full search strategies for all databases, registers and websites, including any filters and limits used. | Table S2 (supplement) |
| Selection process | 8 | Specify the methods used to decide whether a study met the inclusion criteria of the review, including how many reviewers screened each record and each report retrieved, whether they worked independently, and if applicable, details of automation tools used in the process. | Methods – Study Selection |
| Data collection process | 9 | Specify the methods used to collect data from reports, including how many reviewers collected data from each report, whether they worked independently, any processes for obtaining or confirming data from study investigators, and if applicable, details of automation tools used in the process. | Methods – Data Extraction |
| Data items | 10a | List and define all outcomes for which data were sought. Specify whether all results that were compatible with each outcome domain in each study were sought (e.g. for all measures, time points, analyses), and if not, the methods used to decide which results to collect. | Methods – Data Items |
|  | 10b | List and define all other variables for which data were sought (e.g. participant and intervention characteristics, funding sources). Describe any assumptions made about any missing or unclear information. | Methods – Data Items |
| Study risk of bias assessment | 11 | Specify the methods used to assess risk of bias in the included studies, including details of the tool(s) used, how many reviewers assessed each study and whether they worked independently, and if applicable, details of automation tools used in the process. | Methods – Risk of Bias (ROB 2.0) |
| Effect measures | 12 | Specify for each outcome the effect measure(s) (e.g. risk ratio, mean difference) used in the synthesis or presentation of results. | Methods – Statistical Analysis |
| Synthesis methods | 13a | Describe the processes used to decide which studies were eligible for each synthesis (e.g. tabulating the study intervention characteristics and comparing against the planned groups for each synthesis (item #5)). | Methods – Synthesis Methods |
|  | 13b | Describe any methods required to prepare the data for presentation or synthesis, such as handling of missing summary statistics, or data conversions. |  |
|  | 13c | Describe any methods used to tabulate or visually display results of individual studies and syntheses. |  |
|  | 13d | Describe any methods used to synthesize results and provide a rationale for the choice(s). If meta-analysis was performed, describe the model(s), method(s) to identify the presence and extent of statistical heterogeneity, and software package(s) used. |  |
|  | 13e | Describe any methods used to explore possible causes of heterogeneity among study results (e.g. subgroup analysis, meta-regression). | Methods – Subgroup Analysis |
|  | 13f | Describe any sensitivity analyses conducted to assess robustness of the synthesized results. | Methods – Subgroup Analysis |
| Reporting bias assessment | 14 | Describe any methods used to assess risk of bias due to missing results in a synthesis (arising from reporting biases). | Methods – Reporting Bias |
| Certainty assessment | 15 | Describe any methods used to assess certainty (or confidence) in the body of evidence for an outcome. | Methods – Certainty Assessment |
| **RESULTS** | | |  |
| Study selection | 16a | Describe the results of the search and selection process, from the number of records identified in the search to the number of studies included in the review, ideally using a flow diagram. | Results – Study Selection (Fig 1) |
|  | 16b | Cite studies that might appear to meet the inclusion criteria, but which were excluded, and explain why they were excluded. | Results – Study Selection |
| Study characteristics | 17 | Cite each included study and present its characteristics. | Table 1 |
| Risk of bias in studies | 18 | Present assessments of risk of bias for each included study. | Results – Risk of Bias (Fig S1) |
| Results of individual studies | 19 | For all outcomes, present, for each study: (a) summary statistics for each group (where appropriate) and (b) an effect estimate and its precision (e.g. confidence/credible interval), ideally using structured tables or plots. | Results – Figures 2, 3, 4 |
| Results of syntheses | 20a | For each synthesis, briefly summarise the characteristics and risk of bias among contributing studies. | Results – Network Meta-analysis |
|  | 20b | Present results of all statistical syntheses conducted. If meta-analysis was done, present for each the summary estimate and its precision (e.g. confidence/credible interval) and measures of statistical heterogeneity. If comparing groups, describe the direction of the effect. | Results – Figures 2, 3 |
|  | 20c | Present results of all investigations of possible causes of heterogeneity among study results. | Results – Subgroup Analysis |
|  | 20d | Present results of all sensitivity analyses conducted to assess the robustness of the synthesized results. | Results – Statistical Analysis |
| Reporting biases | 21 | Present assessments of risk of bias due to missing results (arising from reporting biases) for each synthesis assessed. | Results – Reporting Bias |
| Certainty of evidence | 22 | Present assessments of certainty (or confidence) in the body of evidence for each outcome assessed. | Results – Certainty of Evidence |
| **DISCUSSION** | | |  |
| Discussion | 23a | Provide a general interpretation of the results in the context of other evidence. | Discussion (first paragraph) |
|  | 23b | Discuss any limitations of the evidence included in the review. | Discussion – Limitations |
|  | 23c | Discuss any limitations of the review processes used. | Discussion – Limitations |
|  | 23d | Discuss implications of the results for practice, policy, and future research. | Discussion – Conclusion |
| **OTHER INFORMATION** | | |  |
| Registration and protocol | 24a | Provide registration information for the review, including register name and registration number, or state that the review was not registered. | Methods – Protocol |
|  | 24b | Indicate where the review protocol can be accessed, or state that a protocol was not prepared. | Methods – Protocol |
|  | 24c | Describe and explain any amendments to information provided at registration or in the protocol. | Not explicitly reported |
| Support | 25 | Describe sources of financial or non-financial support for the review, and the role of the funders or sponsors in the review. | Funding section |
| Competing interests | 26 | Declare any competing interests of review authors. | Conflict of Interest |
| Availability of data, code and other materials | 27 | Report which of the following are publicly available and where they can be found: template data collection forms; data extracted from included studies; data used for all analyses; analytic code; any other materials used in the review. | Data Availability Statement |

# Table S2 Search Strategies

| **Databases** | **Search Details** |
| --- | --- |
| Embase (820) | ('cervical cancer':ab,ti OR 'cervical carcinoma':ab,ti OR 'uterine cervix tumor':ab,ti) AND (recurr*:ab,ti OR persistent:ab,ti OR metastatic:ab,ti OR advanced:ab,ti OR 'neoplasm recurrence':ab,ti OR 'neoplasm metastasis':ab,ti OR 'cancer recurrence':ab,ti) AND (chemotherap*:ab,ti OR 'platinum-based':ab,ti OR paclitaxel:ab,ti OR cisplatin:ab,ti OR carboplatin:ab,ti OR 'immune checkpoint inhibitor*':ab,ti OR immunotherap*:ab,ti OR bevacizumab:ab,ti OR pembrolizumab:ab,ti OR atezolizumab:ab,ti OR cadonilimab:ab,ti OR 'pd-1':ab,ti OR 'pd-l1':ab,ti) AND ('clinicaltrials':ab,ti OR 'phase iii':ab,ti OR 'phase 3':ab,ti OR 'clinical trial':ab,ti) AND [2014-2026]/py |
| Web of Science (598) | ("cervical cancer" OR "cervical carcinoma" OR "uterine cervical neoplasms") (All Fields) and (recurr* OR persistent OR metastatic OR advanced OR "neoplasm recurrence" OR "neoplasm metastasis" OR "cancer recurrence") (All Fields) and (chemotherap* OR "platinum-based" OR paclitaxel OR cisplatin OR carboplatin OR "immune checkpoint inhibitor*" OR immunotherap* OR bevacizumab OR pembrolizumab OR atezolizumab OR cadonilimab OR "PD-1" OR "PD-L1") (All Fields) and 2026 OR 2025 OR 2024 OR 2023 OR 2022 OR 2021 OR 2020 OR 2019 OR 2018 OR 2017 OR 2016 OR 2015 OR 2014 (Year Published) and "ClinicalTrials" OR "phase III" OR "phase 3" OR "clinical trial" (All Fields) |
| PubMed (501) | Search: (((("cervical cancer" OR "cervical carcinoma" OR "uterine cervical neoplasms")) AND ((recurr* OR persistent OR metastatic OR advanced OR "neoplasm recurrence" OR "neoplasm metastasis" OR "cancer recurrence"))) AND ((chemotherap* OR "platinum-based" OR paclitaxel OR cisplatin OR carboplatin OR "immune checkpoint inhibitor*" OR immunotherap* OR bevacizumab OR pembrolizumab OR atezolizumab OR cadonilimab OR "PD-1" OR "PD-L1"))) AND ("ClinicalTrials" OR "phase III" OR "phase 3" OR "clinical trial") Filters: from 2014 - 2025 Sort by: Most Recent |

# Table S3. Proportional Hazards Assumption Testing Results Based on Global Schoenfeld Test

| **Study** | **Outcome** | **Global *p*-value** | **PH Assumption** |
| --- | --- | --- | --- |
| GOG240 | PFS | 0.473 | Satisfied |
| GOG240 | OS | 0.412 | Satisfied |
| KEYNOTE-826 | PFS | 0.179 | Satisfied |
| KEYNOTE-826 | OS | 0.452 | Satisfied |
| BEATcc | PFS | 0.314 | Satisfied |
| BEATcc | OS | 0.921 | Satisfied |
| COMPASSION-16 | PFS | 0.496 | Satisfied |
| COMPASSION-16 | OS | 0.449 | Satisfied |

# Table S4 Search Subgroup Analysis Based on the Bucher Method: Overall Survival

| **Comparison** | **Evidence type** | **HR (95% CI)** | **P value** |
| --- | --- | --- | --- |
| **PD-L1 CPS < 1 subgroup-OS** | | | |
| Pembrolizumab + Chemotherapy ± Bevacizumab  vs Chemotherapy ± Bevacizumab | Direct | 0.87 (0.5, 1.52) | > 0.05 |
| Cadonilimab + Chemotherapy ± Bevacizumab  vs Chemotherapy ± Bevacizumab | Direct | 0.77 (0.44, 1.34) | > 0.05 |
| Pembrolizumab + Chemotherapy ± Bevacizumab  vs Cadonilimab + Chemotherapy ± Bevacizumab | Indirect | 1.13 (0.51, 2.48) | 1.97 |
| **PD-L1 CPS ≥ 1 subgroup-OS** | | | |
| Pembrolizumab + Chemotherapy ± Bevacizumab  vs Chemotherapy ± Bevacizumab | Direct | 0.6 (0.49, 0.74) | < 0.001 |
| Cadonilimab + Chemotherapy ± Bevacizumab  vs Chemotherapy ± Bevacizumab | Direct | 0.69 (0.49, 0.97) | < 0.05 |
| Pembrolizumab + Chemotherapy ± Bevacizumab  vs Cadonilimab + Chemotherapy ± Bevacizumab | Indirect | 0.87 (0.58, 1.29) | 0.491 |
| **Metastatic subgroup-OS** | | | |
| Pembrolizumab + Chemotherapy ± Bevacizumab  vs Chemotherapy ± Bevacizumab | Direct | 0.85 (0.6, 1.21) | > 0.05 |
| Cadonilimab + Chemotherapy ± Bevacizumab  vs Chemotherapy ± Bevacizumab | Direct | 0.73 (0.52, 1.02) | ⁓ 0.05 |
| Pembrolizumab + Chemotherapy ± Bevacizumab  vs Cadonilimab + Chemotherapy ± Bevacizumab | Indirect | 1.16 (0.72, 1.89) | 0.54 |

# Table S5 Search Subgroup Analysis Based on the Bucher Method: Progression-Free Survival

| **Comparison** | **Evidence type** | **HR (95% CI)** | **P value** |
| --- | --- | --- | --- |
| **PD-L1 CPS<1 subgroup-PFS** | | | |
| Pembrolizumab + Chemotherapy ± Bevacizumab  vs Chemotherapy ± Bevacizumab | Direct | 0.95 (0.53, 1.71) | > 0.05 |
| Cadonilimab + Chemotherapy ± Bevacizumab  vs Chemotherapy ± Bevacizumab | Direct | 0.65 (0.42, 1.03) | 0.060 |
| Pembrolizumab + Chemotherapy ± Bevacizumab  vs Cadonilimab + Chemotherapy ± Bevacizumab | Indirect | 1.46 (0.70, 3.06) | 0.313 |
| **PD-L1 CPS ≥ 1 subgroup-PFS** | | | |
| Pembrolizumab + Chemotherapy ± Bevacizumab  vs Chemotherapy ± Bevacizumab | Direct | 0.58 (0.47, 0.71) | < 0.001 |
| Cadonilimab + Chemotherapy ± Bevacizumab  vs Chemotherapy ± Bevacizumab | Direct | 0.62 (0.47, 0.83) | < 0.001 |
| Pembrolizumab + Chemotherapy ± Bevacizumab  vs Cadonilimab + Chemotherapy ± Bevacizumab | Indirect | 0.94 (0.66, 1.33) | 0.708 |
| **Metastatic subgroup-PFS** | | | |
| Pembrolizumab + Chemotherapy ± Bevacizumab  vs Chemotherapy ± Bevacizumab | Direct | 0.71 (0.43, 1.16) | > 0.05 |
| Cadonilimab + Chemotherapy ± Bevacizumab  vs Chemotherapy ± Bevacizumab | Direct | 0.7 (0.54, 0.92) | < 0.05 |
| Pembrolizumab + Chemotherapy ± Bevacizumab  vs Cadonilimab + Chemotherapy ± Bevacizumab | Indirect | 1.01 (0.58, 1.78) | 0.961 |
